# The prognostic significance of lymph node metastasis-related genes in pancreatic adenocarcinoma is associated with immune cell infiltration and ferroptosis

**DOI:** 10.1097/MD.0000000000046435

**Published:** 2025-12-19

**Authors:** Qiang Shu, XiaoLing Liu

**Affiliations:** a Department of Hepatobiliary Surgery, The First People’s Hospital of Neijiang, Neijiang, Sichuan, China; b Department of Nosocomial Infections, Neijiang Hospital of Traditional Chinese Medicine, Neijiang, Sichuan, China.

**Keywords:** ferroptosis, immune infiltration, lymphatic metastasis, pancreatic adenocarcinoma, prognosis

## Abstract

The objective of this study is to investigate the genes associated with lymph node metastasis of Pancreatic adenocarcinoma (PAAD) and their correlation with immune infiltration and ferroptosis. The differentially expressed genes associated with lymphatic metastasis of PAAD were analyzed based on the cancer genome atlas database. The protein-protein interaction network was constructed to screen the hub genes. Functional enrichment analysis was conducted on the hub genes in PAAD with and without lymphoid metastasis. The relationships between the identified genes and both immune cell infiltration and ferroptosis were investigated. LASSO logistic regression analysis was implemented to determine the most relevant genes and construct their risk scores. Multivariate COX regression analysis was conducted based on the genes in the risk score formula. A total of 698 genes were differentially expressed between PAAD with and without lymphatic metastasis, consisting of 153 up-regulated genes and 545 down-Regulated genes. Among the 698 differentially expressed genes, 211 were significantly associated with the overall survival of PAAD patients. The protein-protein interaction network identified 13 hub genes. Only 6 genes, namely CHGA, CHGB, PCSK2, PCSK1N, DLGAP1 and DLGAP3 were down-Regulated in the lymphatic metastasis group. The results of the immune infiltration analysis indicated that the 6 genes were significantly positively correlated with eosinophils, mast cells, pDC, and follicular helper T cells (TFH), and negatively correlated with TH2 cell. Further analysis of the relationship between these 6 genes and ferroptosis revealed that they were positively correlated with the majority of the regulatory factors, namely pyruvate dehydrogenase kinase 4, ALOX15, NCOA4, and BCAT2, and negatively correlated with MGST1 and LCN2. CHGA, PCSK1N, DLGAP1 and DLGAP3 were identified as the 4 most relevant genes through LASSO logistic regression analysis. Multivariate COX regression analysis demonstrated that DLGAP1 was an independent prognostic factor for PAAD. Six hub genes might exert an influence on the initial lymphatic metastasis of PAAD through immune cell infiltration and ferroptosis.

## 1. Introduction

PAAD is a highly detrimental malignancy, projected to become the second leading cause of cancer-related deaths by 2040.^[1]^ Characterized by an invasiveness and dismal prognosis, PAAD is notoriously associated with a high mortality rate, and the 5-year relative survival rate remains a mere 10%.^[2]^ Despite surgical resection being the primary curative option for PAAD, its effectiveness is severely limited due to the frequent recurrence of the disease. There is an urgent need to improve treatment strategies and a deeper understanding of the underlying mechanisms of PAAD progression.

Lymphatic metastasis are frequently and robustly correlated with lower cancer survival.^[3]^ For the majority of cancers, the dissemination of tumor cells via the lymphatic system is the most prevalent route of metastasis. Lymphatic vessels encircle solid tumors and facilitate metastasis by enhancing capillary hyperpermeability and dilation of collecting vessels.^[4]^ Consequently, the existence of Lymph node metastasis (LNM) serves as a crucial indicator of tumor progression and a marker of aggravated tumor stage. LNM serves as a major pathway for the invasion and dissemination of pancreatic cancer, often occurring at early stages of the disease. Pathological LNM in PAAD is recognized as a pivotal prognostic indicator.^[5]^ With the emergence of novel and effective therapeutics, drugs have been developed that exert a significant role in inhibiting tumor lymphatic metastasis by targeting biomarkers. For instance, anlotinib has been proposed to prevent lymphangiogenesis and distant lymphatic metastasis in lung adenocarcinoma by deactivating VEGFR-3 phosphorylation.^[6]^ It has been discovered that it affects LNM in thyroid cancer by modifying tumor immune cell infiltration.^[7]^ Despite the recognized clinical significance of LNM in PAAD, the specific molecular mechanisms driving this process remain poorly elucidated, thereby demanding a predictive biomarker for determining disease progression and prognosis.

Immunotherapy and targeted therapies have been successful in many solid tumors over the last decade. However, these agents have not demonstrated significant benefits for PC patients.^[8]^ The unique pancreatic tumor microenvironment (TME) plays a key role in the failure of most therapeutic strategies, including immunotherapy.^[9,10]^ Therefore, studying the TME is essential to understand the complex interactions among all the actors involved in cancer immune escape mechanisms and treatment resistance, as well as to learn how to exploit the immune system against pancreatic cancer cells.

Based on comprehensive bioinformatics analysis, this study probed into the genes that might influence PAAD lymphatic metastasis and investigated their association with immune infiltration and ferroptosis, with an aim to offer novel insights for the clinical management of PAAD lymphatic metastasis.

## 2. Materials and methods

### 2.1. Data access

**Figure 1.**
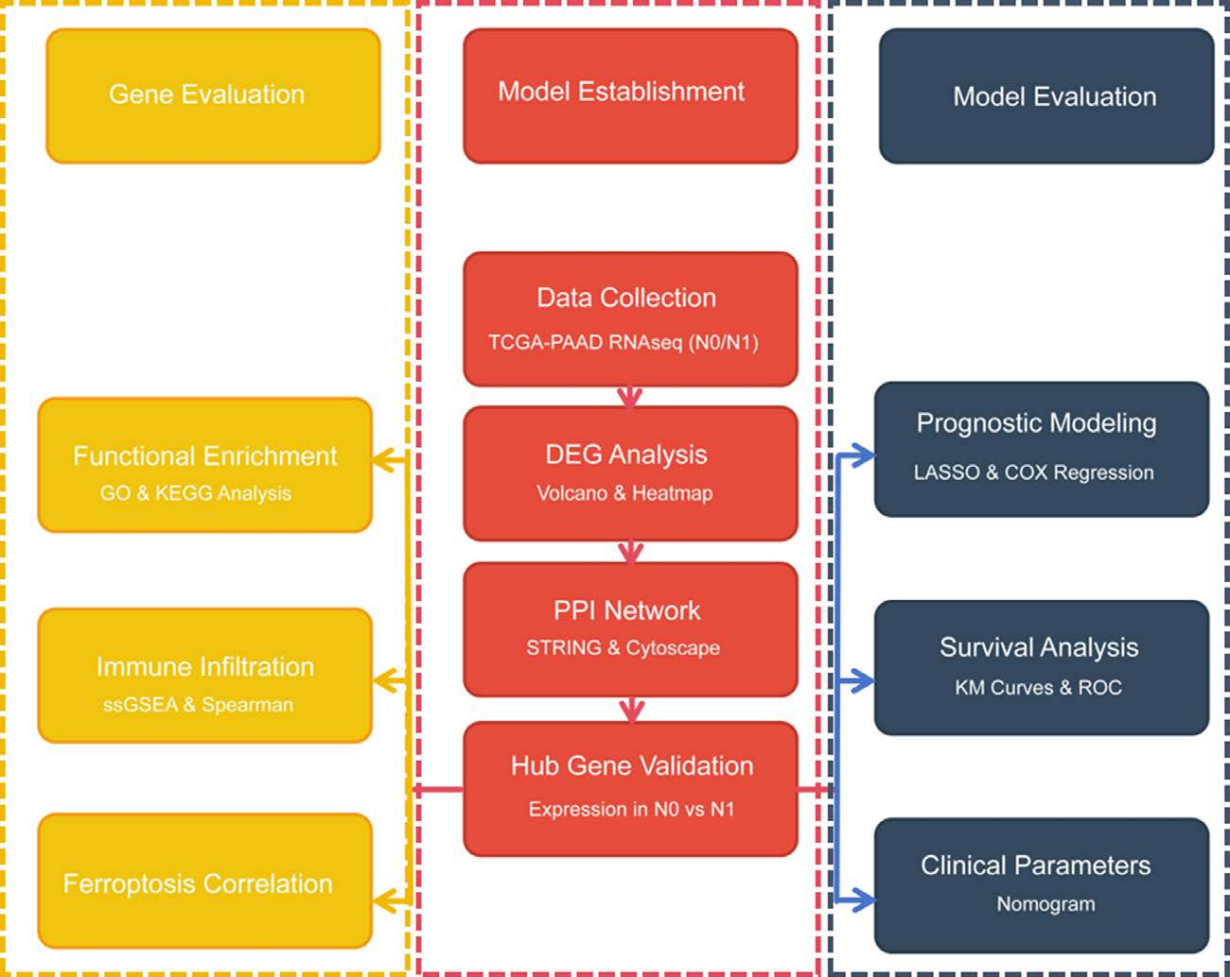
The working flow chart of this study.

**Figure 2.**
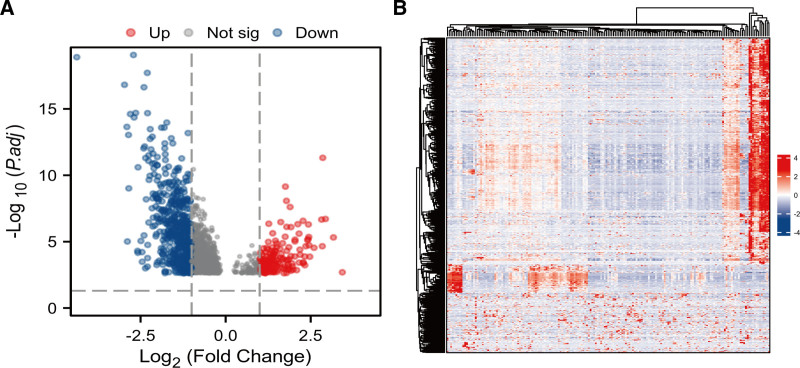
(A) The volcano of DEGs; (B) The heat map of DEGs (red: up-regulate; blue: down-regulate). DEGs = differentially expressed genes.

The workflow of this study is shown in Figure 1. This study utilized the RNA-seq dataset of PAAD from the the Cancer Genome Atlas (TCGA) database(https://www.cancer.gov/ccg/research/genome-sequencing/tcga). A total of 181 samples were encompassed in this study, consisting of 51 without lymphatic metastasis (N0) and 130 with lymphatic metastasis (N1). Additionally, a total of 698 differentially expressed genes (DEGs) in PAAD, consisting of 153 up-regulated genes and 545 down-Regulated genes(Fig. 2).

### 2.2. Construction of volcano map and heat map

The cluster Profiler R software package was employed to analyze the significantly DEG between lymphoid tissues in PAAD. The ggplot2 software package was utilized to visualize the volcano map of the results of differential analysis, and the adjusted threshold values were *P* < .05 and |logFC| > 1. Heat maps were visualized using the Complex Heatmap package.

### 2.3. Construction of the protein–protein interaction network

Refer to STRINGV11 database (https://cn.string-db.org)^[11]^ to analyze the PPI network of DEGs expression. The PPI network was visualized by Cytoscape_v3.10.0 software. MCODE toolkit was used to screen out Hub gene in PPI network.^[12]^

### 2.4. Discrepancies in gene expression and lymphatic metastasis

The PAAD sample data in the TCGA database were classified into 2 groups: those with LNM and those without. Subsequently, the differential mRNA expression was explored using the Limma package of R software. The threshold for screening differential mRNA expression was set when “adjusted *P*-value < 0.05 and |log2 (fold change)| > 1 “.

### 2.5. Assessment of immunoinfiltration

Through a comprehensive literature review, 24 marker genes pertinent to immune cells were identified.^[13]^ The infiltration of these immune cells in PAAD was evaluated using a single sample gene set enrichment analysis (ssGSEA) in R-packet GSVA. The Spearman correlation approach was utilized to analyze the correlation between DEGs expression and immune cells. The range of correlation coefficients was [−1, 1], with negative values signifying negative correlation and positive values signifying positive correlation. The Wilcoxon rank sum test was employed to compare the infiltration of immune cells in PAAD samples with high expression and those with low expression. When *P* < .05, the difference was statistically significant.

### 2.6. Gene set enrichment analysis

The standardized RNA-Seq data originated from TCGA was utilized for GSEA to explore the molecular signaling pathways that various genes might regulate during the progression of PAAD. Based on a 75% threshold, GSEA software (v4.1.0) was adopted to analyze the metabolic pathways and biological processes between the high and low expression of distinct genes. GESA parameter settings selected the c2KEGG gene set (c2.cp). kegg, V7.5.symbols.gmt) and set the number of permutations of the gene set to 1000. The enrichment results with a standardized *P* < .05 and an error discovery rate < 0.25 were statistically significant.

### 2.7. Enrichment analysis

Gene ontology (GO) enrichment analysis (BP: biological process; CC: cellular component; MF: molecular function) and Kyoto encyclopedia of genes and genomes (KEGG) pathway analysis were conducted on the screened lymphatic metastasis–associated genes using R package cluster profiles. A cutoff value of *P* < .05 was used to enrich functional categories and pathways.

### 2.8. Prognostic modeling

LASSO regression methods was conducted on 6 genes significantly associated with lymphatic metastasis, and 4 genes that were most associated with prognosis were identified, along with the construction of 4-gene signature. Subsequently, a risk score for each patient was calculated based on the regression coefficients and corresponding expression values of the genes in the characteristics. The risk score is computed using the formula:


$$\begin{array}{l} \begin{array}{lll} & Risk\;score=expression\;of\;Gene\;1*\beta \;1+expression\;of\;Gene\;2*\beta \;2\;+\; & \cdots +expression\;of\;Gene\;n*\;\beta \;n.\; \end{array} \end{array}$$

Patients were classified into high-risk and low-risk groups based on the median risk score. Overall survival (OS) was analyzed through the Kaplan–Meier method, followed by the implementation of the logarithmic rank test. The sensitivity and accuracy of the signature were verified by receiver operating characteristic curves (ROC) using the Survival ROC package in R. Differences were considered statistically significant when *P* < .05.

## 3. Result

### 3.1. Analysis of DEGs in lymphatic metastasis and non-lymphatic metastasis

The DEGs in PAAD with and without lymphatic metastasis were analyzed based on the TCGA database. The results indicated that there were 698 DEGs in PAAD with and without lymphatic metastasis, consisting of 153 up-regulated genes and 545 down-Regulated genes. The volcano and the heat map of DEGs are presented in Figure 2.

### 3.2. Construction of the PPI network

**Figure 3.**
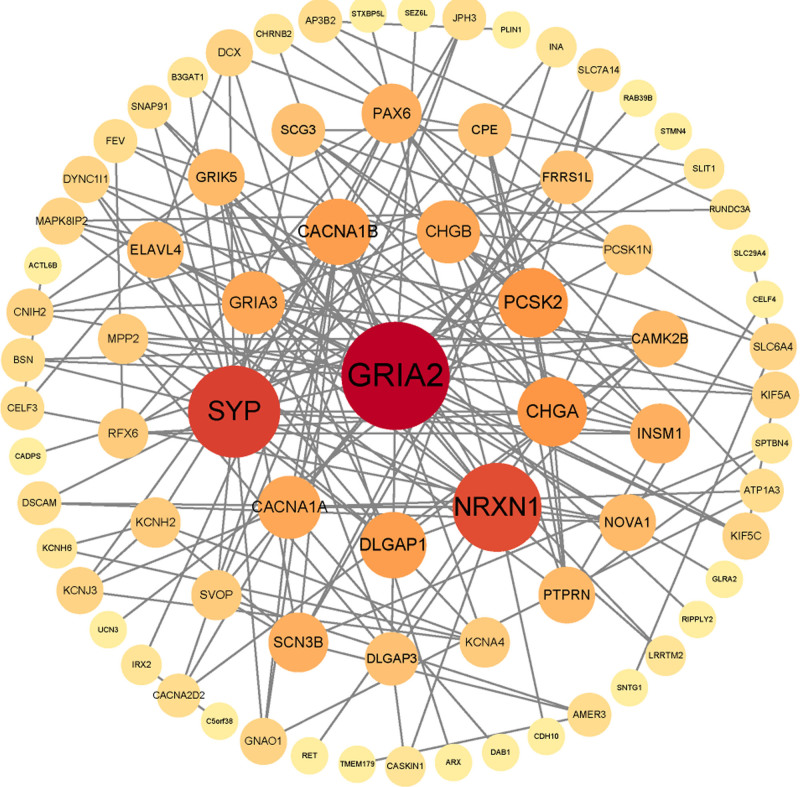
The PPI networks of 211 DEGs. DEGs = differentially expressed genes, PPI = protein–protein interaction.

Among the 698 DEGs, 211 were significantly associated with the overall survival of PAAD patients and constructed PPI networks (Fig. 3). The top 20 hub genes were screened via the MCC method and the DMNC method, and a total of 13 overlapping hub genes were identified. They are respectively CHGA, CHGB, PCSK2, CPE, SCG3, PCSK1N, DLGAP1, DLGAP3, MPP2, GRIK5, GRIA3, PTPRN and CAMK2B.

### 3.3. Expression of 6 hub genes in PAAD with and without lymphatic metastasis

**Figure 4.**
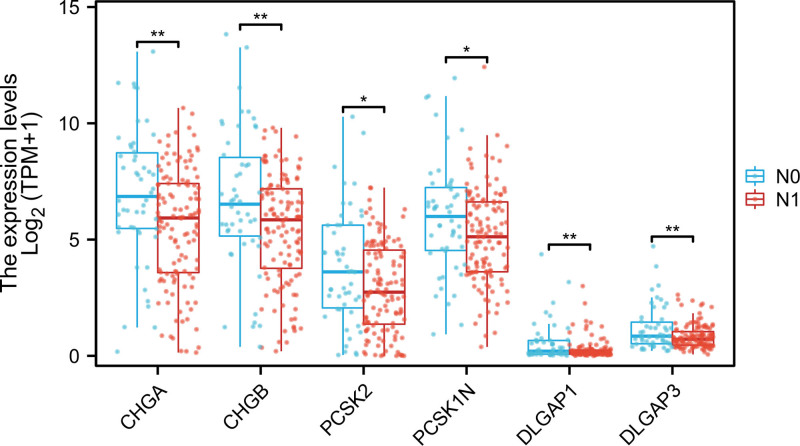
The expression of the 6 hub genes in PAAD with and without lymphatic metastasis.* indicates *P* < .05, ** indicates *P* < .01. PAAD = pancreatic adenocarcinoma.

**Figure 5.**
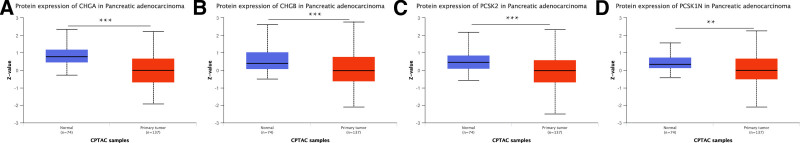
The protein expression of the hub genes in PAAD.** indicates *P* < .01, *** indicates *P* < .001. PAAD = pancreatic adenocarcinoma.

The construction of the PPI network identified 13 significant hub genes. Subsequently, the analysis of the expression of these genes in PAADs indicated that there were substantial differences in the expression of CHGA, CHGB, PCSK2, PCSK1N, DLGAP1 and DLGAP3 genes in PAAD with and without lymphatic metastasis, and their expressions were down-regulated in the lymphatic metastasis group (Fig. 4). The protein expression retrieved from the CPTAC are presented in the Figure 5.

### 3.4. Gene set enrichment analysis

**Figure 6.**
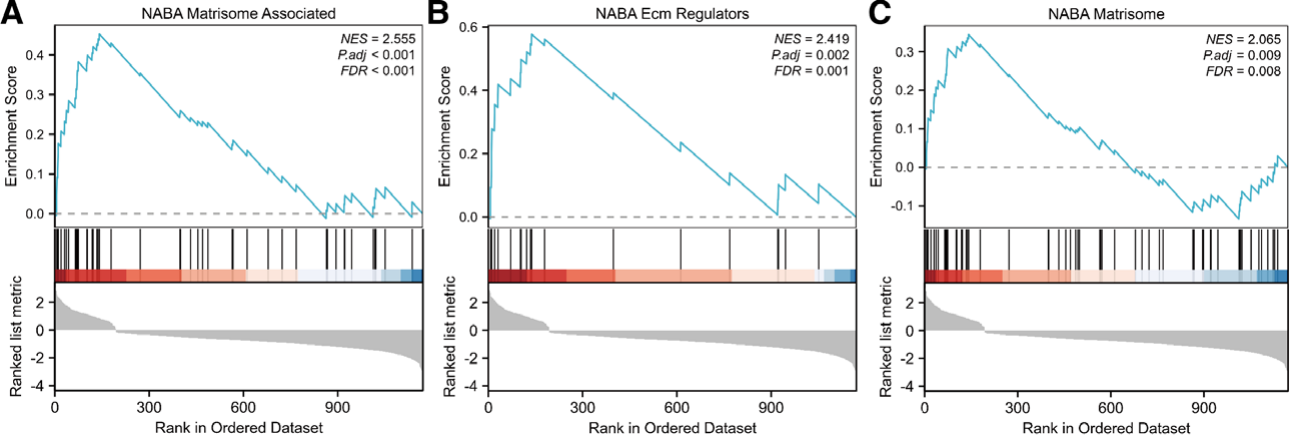
The GSEA analysis of 698 DEGs. (A) matrisome; (B) ECM-regulators; (C) matrisome-associated. DEGs = differentially expressed genes, ECM = extracellular matrix, GSEA = gene set enrichment analysis.

The GSEA analysis of 698 DEG in PAAD with and without lymphatic metastasis revealed that the DEG were mainly clustered in 3 pathways (Fig. 6A–C): matrisome, ECM-regulators and matrisome-associated.

### 3.5. GO and KEGG analysis

**Figure 7.**
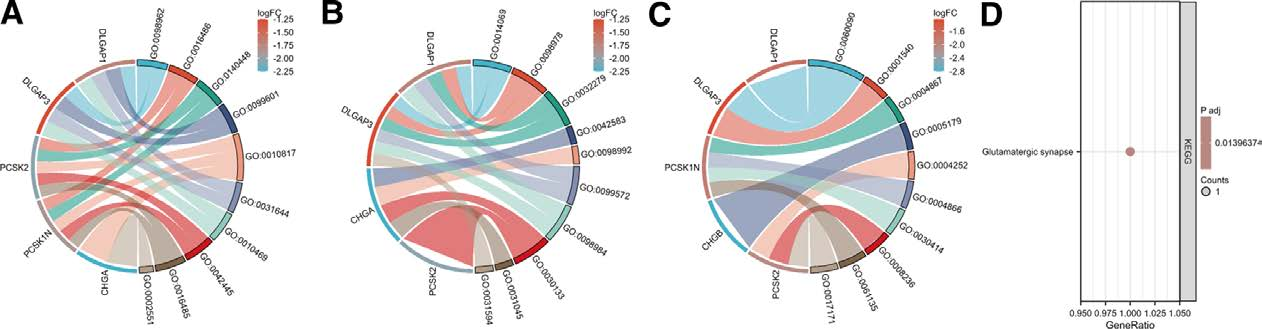
GO analysis and KEGG analysis of the 6 hub genes. (A) Circle plot of BP analysis; (B) Circle plot of CC analysis; (C) Circle plot of MF analysis; (D) KEGG analysis. GO = gene ontology, KEGG = Kyoto encyclopedia of genes and genomes.

To comprehend the molecular mechanism of lymphatic transfer-related genes, we conducted enrichment analysis of these 6 DEGs. The outcomes of GO analysis indicate that these DEGs play significant roles in glutamatergic synapse, mast cell chemotaxis, peptide hormone processing, chromaffin granule, regulation of signaling receptor activity and transport vesicle (Fig. 7A–C). The KEGG analysis demonstrated that this DLGAP1 gene is of great significance in the glutaminergic synaptic pathway (Fig. 7D).

### 3.6. The relationship between 6 DEGs and immune infiltration in PAAD

**Figure 8.**
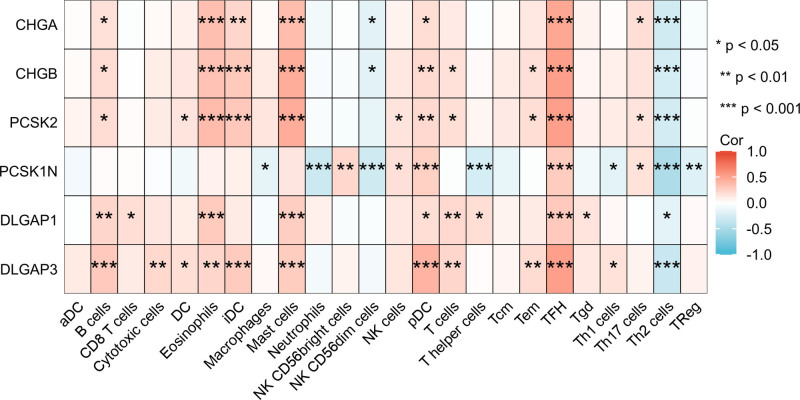
Six genes respectively in relation to the level of immune cell infiltration. *P <.05, **P <.01,***P <.001.

Enrichment analysis demonstrated that genes exert a crucial role in encoding and regulating the extracellular matrix^[14]^ and mast cell chemotaxis, which are intimately associated with the immune response. Hence, the association between 6 DEGs in PAAD and immune infiltration was examined. The outcomes of the ssGSEA analysis demonstrated that the 6 DEGs were significantly positively correlated with eosinophils, mast cells, plasmacytoid dendritic cells (pDC), and follicular helper T cells, while being negatively correlated with helper T2 cells, indicating that these genes play a crucial role in the immune infiltration of PAAD(Fig. 8).

### 3.7. The relationship between 6 DEGs and ferroptosis in PAAD

**Figure 9.**
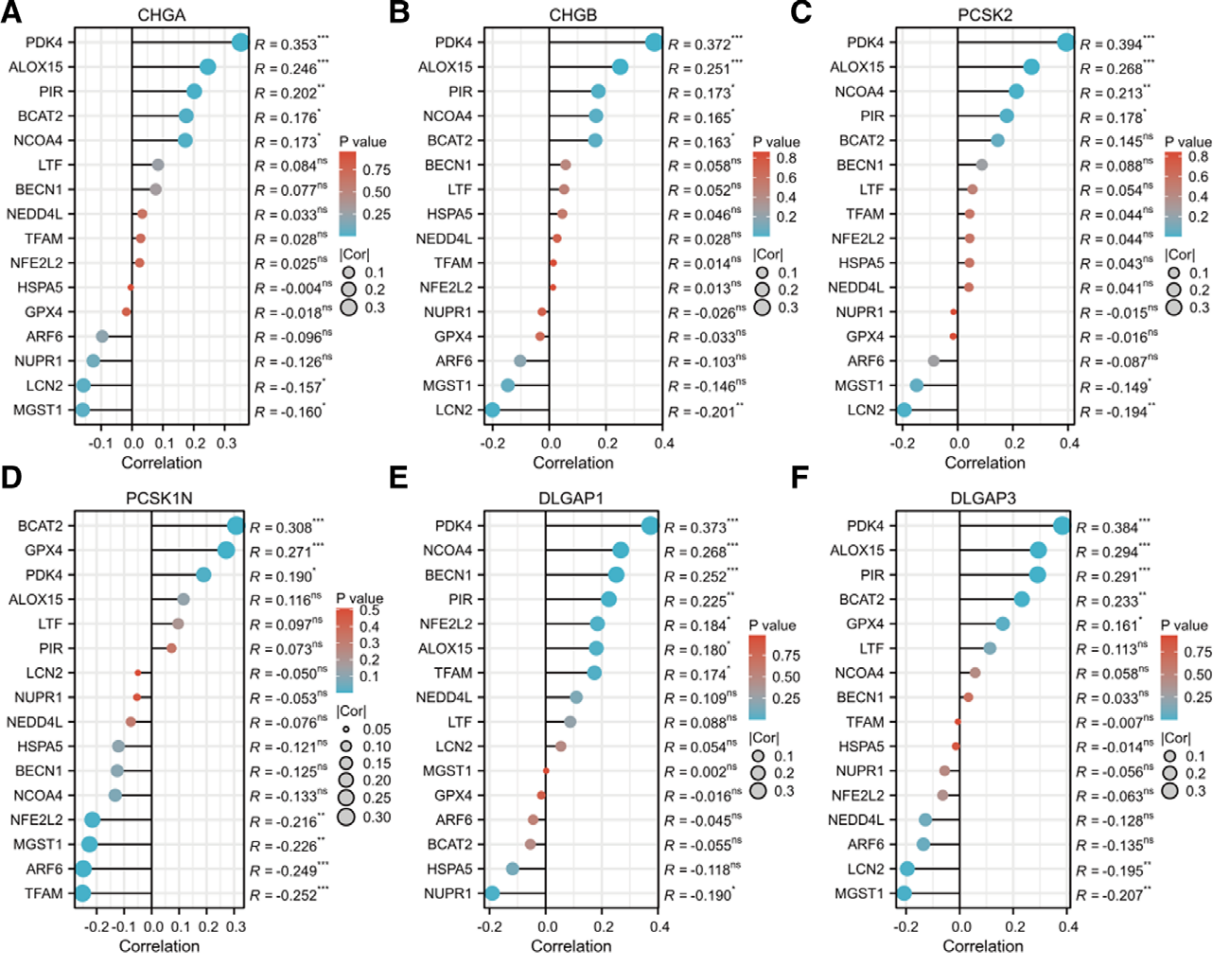
(A–F) The lollipop plot of the relationship between 6 DEGs and ferroptosis. DEGs = differentially expressed genes.

Through enrichment analysis, 6 DEGs were predominantly enriched in the glutamatergic synaptic pathway, which regulates glutamine metabolism. Previous studies have affirmed that glutamine deprivation induces ferroptosis in pancreatic cancer cells.^[15]^ We further investigated the correlation between the aforementioned DEG and ferroptosis, which was closely associated with the majority of ferroptosis regulators in pancreatic adenocarcinoma^[16]^(Figs. 9A-F).

### 3.8. Prognostic modeling

**Figure 10.**
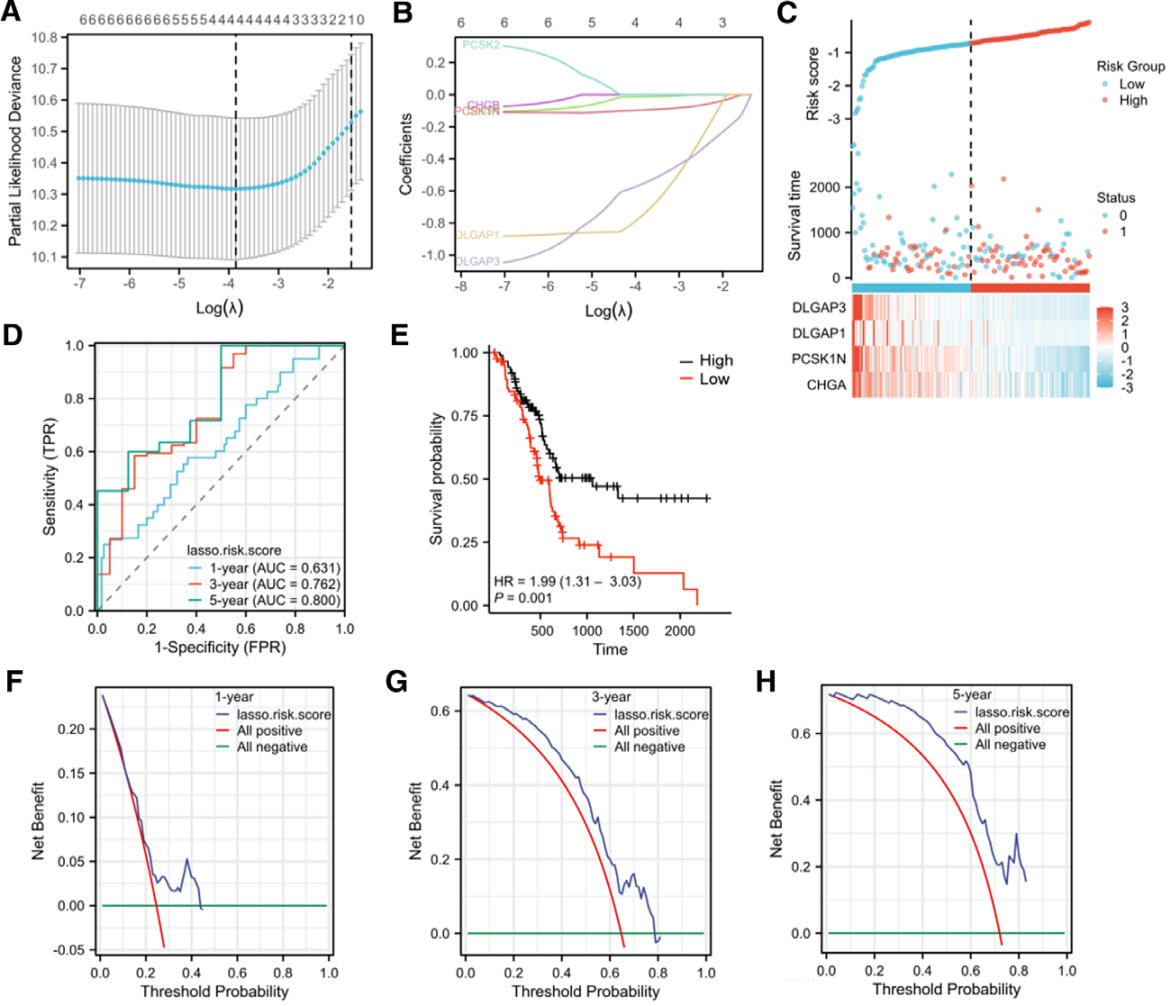
Prognostic risk modeling. (A): LASSO regression cross-validation map of related genes; (B): LASSO regression coefficient diagram of related genes; (C): Risk score, survival time, and survival status in the TCGA dataset; (D) 1-, 3-, and 5-yr ROC curves of the risk model; (E) K-M curve for high-risk and low-risk patients in the risk model; (F–H) DAC curves of the risk model of 1, 3, 5 yr. ROC = receiver operating characteristic.

LASSO logistic regression analysis was conducted on 6 genes expression matrices in the PAAD cohort, and the risk scores of 4 gene constructs significantly related to lymphatic metastasis were determined (Fig. 10).

### 3.9. The risk score is calculated by the formula:


$$\begin{array}{l} \begin{array}{lll} & Riskscore=\left(-0.3537\right)*EVA1A+\left(-0.2885\right)*SERPINA1+\; & \left(0.3839\right)*FN1+\left(0.1075\right)*TNC+\left(0.116\right)*MXRA8\; \end{array} \end{array}$$

Subsequently, we examined the distribution of K-M survival curves of the risk model in the TCGA dataset. The findings indicated that the survival status of high-risk patients was significantly poorer than that of low-risk patients [HR = 1.99 (1.31–3.03), *P* = .001]. The 1-year, 3-year and 5-year area under curve values were respectively 0.631, 0.762 and 0.800. The decision curve analysis of the risk model reveals that it has excellent clinical utility.

**Figure 11.**
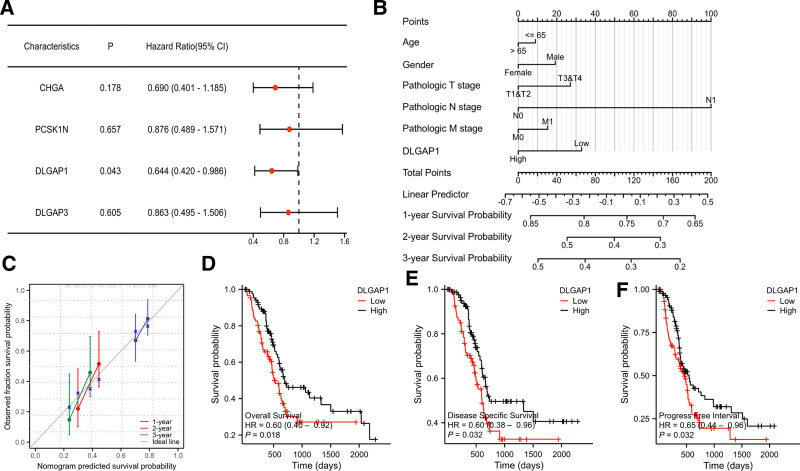
(A): Multifactor COX regression analysis for 4 genes within the risk-based scoring formula; (B): The Nomogram of clinically relevant factors; (C): Calibration curve of Nomogram. (D–F): K-M curves for overall survival, disease-specific survival, and progression-free interval regarding the expression level of DLGAP1.

Furthermore, we executed a multifactor COX regression analysis for 4 genes within the risk-based scoring formula, substantiating that DLGAP1 was an independent prognostic factor (Fig. 11A). For the establishment of a Nomogram of clinically relevant factors incorporating the DLGAP1 gene, the results disclose that the expression level of DLGAP1 conspicuously affects the prognosis of patients (Fig. 11B). The calibration curve predicted the 1-year, 2-year and 3-year survival prognosis, suggesting that the bias correction line was close to the ideal curve, and the predicted value of the curve was in excellent agreement with the actual results (Fig. 11C). DLGAP1 expression levels were categorized into high and low expression groups in accordance with the median. Overall survival, disease-specific survival and progression-free interval were analyzed by means of the K-M method. The findings demonstrated that for patients with low expression of DLGAP1, those with the high expression of DLGAP1 had a more favorable overall survival, disease-specific survival and progression-free interval (Fig. 11D–F).

**Figure 12.**
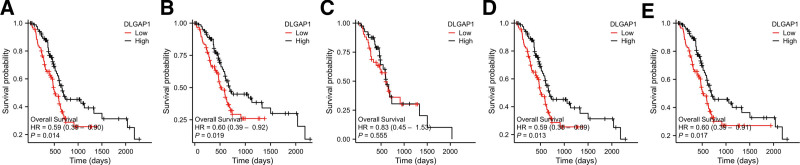
(A–E): K-M curves for T stage, N stage, M stage, pathologic stage, and histological grade regarding the expression level of DLGAP1.

Additionally, the expression level of DLGAP1 were classified into high and low expression groups based on the median, and the K-M method was utilized for subgroup analysis of clinically relevant factors. The findings indicated that in the high expression group, PAAD patients exhibited better TNM stage, pathologic stage, and histological grade. With the exception of the M stage (HR = 0..83 [0.45–1.53], *P* = .555), the remaining factors, namely the T stage (HR = 0.59 [0.39–0.90], *P* = .014), the N stage (HR = 0.60 [0.39–0.92], *P* = .019), the pathologic stage (HR = 0.59 [0.38–0.90], *P* = .013), and the histological grade (HR = 0.60 [0.39–0.91], *P* = .017), were statistically significant (Fig. 12A–E).

### 3.10. Survival analysis on 6 DEGs

**Figure 13.**
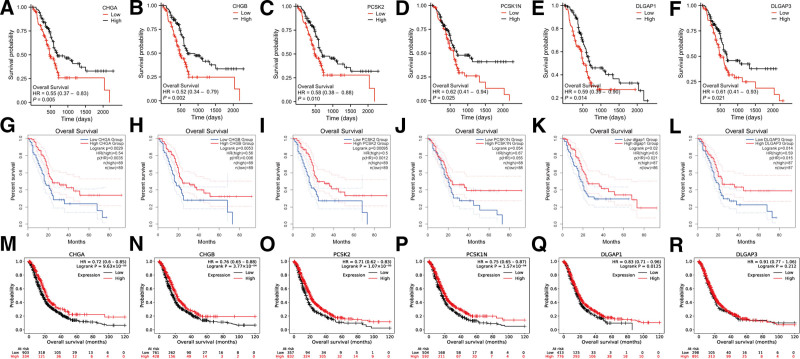
(A–F): K-M curves for 6 hub genes regarding the expression level of DLGAP1 in the TCGA database; (G–L): K-M curves for 6 hub genes regarding the expression level of DLGAP1 in In the K-M plotter database; (M–R): K-M curves for 6 hub genes regarding the expression level of DLGAP1 in the GEPIA2 databases.

In the K-M plotter database (http://kmplot.com/analysis/) and GEPIA2 database (http://gepia2.cancer-pku.cn/) respectively, the retrieval was conducted in the databases to draw the expression levels of 6 DEGs and the survival of patients with pancreatic cancer (Fig. 13G–R). Consistent with our results, the higher expression levels of the 6 DEGs were significantly associated with a better survival prognosis (Fig. 13A–F).

## 4. Discussion

Tumor metastasis pertains to the dissemination of cancer cells from the primary tumor to the circulatory system and the colonization of distant organs. It typically encompasses hematogenous metastasis and lymphatic metastasis, and constitutes one of the most crucial factors in tumor progression.^[17]^ Lymphatic metastasis is directly correlated with distant recurrence and overall survival in the majority of tumors.^[18]^ PAAD constitutes a highly aggressive tumor type, and in the process of metastasis, cancer cells mainly disseminate through the lymph nodes.^[19]^ Lymphatic metastasis, which frequently occurs in the early phases of the disease, is an independent predictor for PAAD survival.^[20]^ Therefore, it is indispensable to identify biomarkers capable of predicting the early lymphatic metastasis of PAAD.

In this study, we detected 698 DEG, among which 153 were upregulated and 545 were downregulated between the groups of samples with and without lymphatic metastasis. Of the 698 DEG, 211 were significantly associated with the overall survival of PAAD patients. We constructed the PPI network involving 211 genes and screened out a total of 6 hub genes associated with lymphatic metastasis: CHGA, CHGB, PCSK2, PCSK1N, DLGAP1 and DLGAP3. CHGA is a 49 kDa glycoprotein gene located on chromosome 14 that is primarily expressed by endocrine and neuroendocrine cells.^[21]^ CHGA can be secreted into the blood in its entirety or in small fragments after fragmentation. Although the precise function of CHGA peptides is not fully understood, they are believed to play a key role in fat metabolism, immune properties, and reproduction. Chromogranin A has been proposed as promising biomarkers for Colorectal Cancer detection in the early stages.^[22]^ CHGB is one of the 2 major soluble proteins in the chromophobe granules of the adrenal medulla.^[23]^ CHGB, instrumental in immune modulation, exhibits deviant gene expression across myriad tumor varieties, with its augmented expression being intrinsically linked to metastatic events. It may be a prognostic marker in neuroendocrine tumors, correlates with survival in colon cancer, paragangliomas.^[24,25]^ PCSK2 encodes a protein-degrading enzyme responsible for activating inactive prohormones into active peptides. Ma and Luo reported in previous esophageal cancer studies that PCSK2, when creating a gene signature with differential expression genes, can negatively influence the prognosis of esophageal squamous cell carcinoma patients.^[26]^ Additionally, Li et al^[27]^ reported that PCSK2 is associated with the survival of esophageal cancer patients as an immune-related gene.^[28]^ The granule protein family member PCSK1N, also known as ProSAAS, is a protein produced almost entirely by a wide variety of endocrine, neuronal and neuroendocrine cells.^[29,30]^ The proteolytic neuropeptide PEN derived from the precursor ProSAAS has been identified as a selective, high affinity endogenous ligand for the orphan receptor GPR83. Both of them show regional specific expression in neuroendocrine tissues and may be used as a target for the treatment of neurological and immune diseases.^[31]^ DLGAP1, also known as GKAP, SAPAP1, or DAP-1, belongs to the SAPAP family and is a postsynaptic protein, which plays an important role in the formation of synapses and the signaling of nerve cells.^[32]^ DLGAP1 is polarity protein, which regulates cell development, maintenance of cell polarity, damage repair, and tissue integrity.^[33]^ Changes in cell polarity may promote tumorigenesis, epithelial-mesenchymal transition, and cell invasion and metastasis.^[34]^ LI demonstrated for the first time that EMT can be elicited by upregulating the expression of DLGAP1, thereby facilitating the migration and invasion of Glioblastoma cells.^[35]^ The protein encoded by the DLGAP3 gene is a cytoskeleton protein replete with domains and is closely associated with interneuronal communication and synaptic plasticity. Studies have demonstrated that DLGAP3 participates in synaptic formation and synaptic transmission through interaction with other proteins and is indispensable for the normal development and functional maintenance of the nervous system.^[36]^

In this paper, it was discovered that the genes of CHGA, CHGB, PCSK2, PCSK1N, DLGAP1 and DLGAP3 were conspicuously down-regulated in the lymphatic metastasis of PAAD. The lymphatic system serves as an interface between innate and adaptive immunity and is competent in actively communicating and sensing inflammatory stimuli from the periphery.^[37]^ Lymph nodes are also common sites for tumor metastasis, and cancer cells within the lymph nodes can shape their interactions with the host immune system by regulating the invasion and reactivity of immune cells.^[38]^ The extracellular matrix (ECM) is a dynamic tissue support network constituted by fibrin, glycosaminoglycan, proteoglycan and mucus. The molecular, physical and mechanical attributes of ECM modulate the motility, survival and functionality of immune cells. It is increasingly recognized that this network bears an interdependent relationship with immune cells.^[14]^ This might imply that lymphatic metastasis could be closely associated with the infiltration of immune cells. Our GSEA analysis and GO analysis reveal that differential genes exert a significant role in encoding and regulating the extracellular matrix and mast cell chemotaxis, which are intimately related to immune responses. It is believed that activated eosinophils exert an influence on carcinogenesis and tumor progression through various mechanisms within the tumor microenvironment. A meta-analysis study demonstrated that eosinophilic infiltration was significantly correlated with the enhancement of overall survival in esophageal and colorectal cancer. Moreover, eosinophilic infiltration was inversely associated with LNM.^[39]^ Elevated concentrations of follicular helper T cells are correlated with a decreased number of lymphatic metastases, and lower levels of TFH are associated with superior overall survival in patients with gastric cancer, which might exert a crucial role in suppressing the immune response to gastric cancer.^[40]^ TH2 cells and innate lymphoid cells 2 are capable of stimulating tumor growth through the secretion of pro-tumor cytokines like IL-4, IL-5 and IL-13. Studies have indicated that elevating the expression of IL-33 in pancreatic ductal adenocarcinoma (PDAC) cells can diminish the infiltration of TH2 and innate lymphoid cells 2 and enhance the survival rate of PDAC patients.^[41]^ It has also been evidenced that the activation of mast cells can mitigate melanoma metastasis.^[42]^

Therefore, the correlation between 6 genes in PAAD and immune cells was examined. The results of the ssGSEA analysis indicated that these 6 genes were significantly and positively correlated with eosinophils, TFH, mast cells and plasmacytoid dendritic cells, while being negatively correlated with Th2 cells. The 6 genes are significantly downregulated in the lymphatic metastasis of PAAD, and a high level of gene expression is associated with a more favorable survival prognosis. These findings suggest that the 6 genes exert a crucial inhibitory role on immune cell infiltration in PAAD. Although there is no conclusive study on their role in PAAD immune infiltration, it has been shown that variations in their levels have an influence on the level of immune cells and thereby on the disease progression. Although pancreatic cancer shows obvious characteristics of immune cell infiltration, the predominant feature remains immunosuppression.^[43]^ Whether the expression level of the aforementioned genes can overcome this immunosuppression and improve the survival prognosis of pancreatic adenocarcinoma patients demands further investigation.

In the GO and KEGG analyses, 6 differential genes were ascertained to be preponderantly concentrated within the glutaminergic synaptic pathway and govern the metabolism of glutamine. It has been manifested that glutamine deprivation is capable of inducing ferroptosis in pancreatic cancer cells.^[15]^ Henceforth, we executed a more comprehensive and profound analysis of the correlation between the aforementioned DEGs and ferroptosis.

Ferroptosis represents a novel modality of programmed cell death that is initiated by the lethal accumulation of lipid peroxides catalyzed by biologically active iron within the cell. As of now, the impacts of diverse ferroptosis regulators have been elucidated to impinge on the ferroptotic sensitivity.^[16]^ Pyruvate dehydrogenase kinase 4 (PDK4) is a crucial enzyme that governs the activity of the pyruvate dehydrogenase complex and serves as a key regulator of pyruvate oxidation and glucose homeostasis. PDK4 inhibits ferroptosis by impeding pyruvate dehydrogenase-dependent pyruvate oxidation and fatty acid synthesis.^[44]^ For patients afflicted with ovarian cancer, drugs that target inherent metabolic vulnerability (such as GLUT1 inhibitors, PDK4 inhibitors, or glutaminase inhibitors) have a tendency to induce ferroptosis in cancer cells.^[45]^ ALOX15, also denominated as arachidonic acid 15-lipoxygenase, potentiates ferroptosis through the conversion of intracellular unsaturated lipids into oxidized lipid intermediates and represents an essential target for ferroptosis.^[46]^ Research endeavors have manifested that the expression of ALOX15 constitutes an independent prognostic determinant for cervical cancer. The low expression of ALOX15 restrains ferroptosis within cervical cancer cells and is positively affiliated with the auspicious prognosis of cervical cancer.^[47]^ Nuclear receptor coactivity 4 (NCOA4)-mediated ferritin autophagy exerts an increasingly essential role in maintaining intracellular iron homeostasis by facilitating ferritin transport and iron release. The knockdown of NCOA4 conspicuously inhibited the reduction of ferritin, lowered the level of intracellular free iron, and alleviated radiation-induced ferroptosis in intestinal epithelial cells.^[48]^ Branchchain amino acid aminotransferase 2 (BCAT2) acts as a novel inhibitor of ferroptosis. We have verified that BCAT2, as a crucial enzyme implicated in mediating sulfur amino acid metabolism, regulates intracellular glutamate levels, and its specific expression shields liver cancer and pancreatic cancer cells from ferroptosis. Conversely, inhibiting the activity of BCAT2 triggers ferroptosis in cancer cells.^[49]^ Microsomal glutathione S-transferase 1 (MGST1), a selenium-independent glutathione peroxidase, plays a crucial role in safeguarding the endoplasmic reticulum and the outer membrane of mitochondria from lipid peroxidation, thereby protecting cells from ferroptosis.^[50]^ The deletion of the MGST1 gene facilitates ferroptosis in pancreatic cancer cells, and the reexpression of MGST1 restores the resistance of NFE2L2 knockdown cells to ferroptosis. A portion of MGST1 reduces lipid peroxidation through binding to ALOX5 and inhibits the ferroptosis of carcinogenic cells.^[51]^ Lipid carrier protein 2 (LCN2), also referred to as neutrophil gelatinase-associated lipid carrier protein, has been demonstrated to function as an iron regulator under physiological and inflammatory circumstances and is a crucial iron metabolic factor. LCN2 serves as a mediator in numerous diseases related to cachexia, inducing ferroptosis and depletion of adipose and muscle tissues in lung cancer cachexia.^[52]^ The knockdown of iron metabolism genes FPN and LCN2 via CRISPR/Cas13a and microRNA gave rise to significant ferroptosis in multiple hematological and solid tumor cancer cells.^[53]^

Through the analysis of the relationship between the 6 genes and ferroptosis, it was ascertained that they were positively correlated with the majority of ferroptosis regulators such as PDK4, ALOX15, NCOA4, BCAT2 and negatively correlated with MGST1 and LCN2. It can be discerned from the outcomes of this study that the aforementioned 6 genes might facilitate lymphatic metastasis of pancreatic cancer by suppressing the ferroptosis pathway. Despite the absence of a definite study regarding their role in ferroptosis of pancreatic cancer, alterations in their levels have been demonstrated to exert an influence on the regulation of ferroptosis and thereby on the advancement of the disease. Nevertheless, the specific mechanism demands further investigation.

Ultimately, we executed LASSO logistic regression analysis on the 6 gene expression matrices within the TCGA database in light of the presence or absence of LNM, and ultimately discerned the 4 most pertinent genes (CHGA, PCSK1N, DLGAP1 and DLGAP3) for constructing their risk scores. Additionally, the multivariate Cox regression analysis of the 4 genes identified through the risk score formula suggested that DLGAP1 was an independent prognostic indicator for PAAD. A Nomogram encompassing clinically relevant factors inclusive of the DLGAP1 gene was established, and the results manifested that the expression level of DLGAP1 and lymphatic metastasis exerted a significant influence on the prognosis of PAAD patients. The impacts of the level of DLGAP1 expression on the overall survival, disease-specific survival, and progression-free interval of PAAD patients were analyzed by means of the K-M method. The findings disclosed that PAAD patients in the high expression of DLGAP1 group enjoyed more favorable overall survival, disease-specific survival, and progression-free interval. Subgroup analysis of clinical relevant factors in PAAD patients was conducted. Results indicated that the TNM stage, pathological stage, and histological grade of PAAD patients with high expression of DLGAP1 were superior to those with low expression. These results can be construed as indicating that the expression level of DLGAP1 is intimately linked to the prognosis of PAAD lymphatic metastasis.

Certainly, our study inevitably possesses certain constraints. TCGA is a frequently refreshed public database, the sample size and data volume are constrained, and the clinicopathological information is deficient. This might engender some potential errors or biases. Additional data could subsequently be included to optimize the model. Given that the study is predicated on bioinformatics analysis, in vivo and in vitro experimental validations are absent, we will persevere in this study.

In summary, our findings reveal that 6 genes are associated with lymphatic metastasis of PAAD. The 6 genes are significantly positively correlated with eosinophils, mast cells, pDC, and TFH, and negatively correlated with Th2 cells. The 6 genes were positively correlated with PDK4, ALOX15, NCOA4, BCAT2 and negatively correlated with MGST1 and LCN2 in PAAD. This implies that these 6 genes might contribute to the lymphatic metastasis of PAAD by inhibiting immune cell infiltration and the ferroptosis process. Furthermore, DLGAP1 is closely associated with the prognosis of PAAD lymphatic metastasis, providing a potential therapeutic orientation for targeting to impede the advancement of PAAD.

## Author contributions

**Conceptualization:** Qiang Shu.

**Data curation:** Qiang Shu, XiaoLing Liu.

**Formal analysis:** Qiang Shu, XiaoLing Liu.

**Methodology:** Qiang Shu.

**Project administration:** Qiang Shu.

**Software:** Qiang Shu.

**Supervision:** Qiang Shu.

**Visualization:** Qiang Shu.

**Writing – original draft:** Qiang Shu, XiaoLing Liu.

**Writing – review & editing:** Qiang Shu.
